# Innovative multidimensional gait evaluation using IMU in multiple sclerosis: introducing the semiogram

**DOI:** 10.3389/fneur.2023.1237162

**Published:** 2023-09-15

**Authors:** Cyril Voisard, Nicolas de l'Escalopier, Aliénor Vienne-Jumeau, Albane Moreau, Flavien Quijoux, Flavie Bompaire, Magali Sallansonnet, Marie-Laure Brechemier, Irina Taifas, Camille Tafani, Eve Drouard, Nicolas Vayatis, Damien Ricard, Laurent Oudre

**Affiliations:** ^1^Université Paris Saclay, Université Paris Cité, Ecole Normale Supérieure Paris Saclay, Centre National de la Recherche Scientifique, Service de Santé des Armées, Institut National de la Santé et de la Recherche Médicale, Centre Borelli, Gif-sur-Yvette, France; ^2^Service de Neurologie, Service de Santé des Armées, Hôpital d'Instruction des Armées Percy, Clamart, France; ^3^Université Paris Cité, Université Paris Saclay, Ecole Normale Supérieure Paris Saclay, Centre National de la Recherche Scientifique, Service de Santé des Armées, Institut National de la Santé et de la Recherche Médicale, Centre Borelli, Paris, France; ^4^Service de Chirurgie Orthopédique, Traumatologique et Réparatrice des Membres, Service de Santé des Armées, Hôpital d'Instruction des Armées Percy, Clamart, France; ^5^Ecole du Val-de-Grâce, Service de Santé des Armées, Paris, France

**Keywords:** gait quantification, gait disorders, multiple sclerosis, wearable inertial sensors, inertial measurement unit

## Abstract

**Background:**

Quantifying gait using inertial measurement units has gained increasing interest in recent years. Highly degraded gaits, especially in neurological impaired patients, challenge gait detection algorithms and require specific segmentation and analysis tools. Thus, the outcomes of these devices must be rigorously tested for both robustness and relevancy in order to recommend their routine use. In this study, we propose a multidimensional score to quantify and visualize gait, which can be used in neurological routine follow-up. We assessed the reliability and clinical coherence of this method in a group of severely disabled patients with progressive multiple sclerosis (pMS), who display highly degraded gait patterns, as well as in an age-matched healthy subjects (HS) group.

**Methods:**

Twenty-two participants with pMS and nineteen HS were included in this 18-month longitudinal follow-up study. During the follow-up period, all participants completed a 10-meter walk test with a U-turn and back, twice at M0, M6, M12, and M18. Average speed and seven clinical criteria (sturdiness, springiness, steadiness, stability, smoothness, synchronization, and symmetry) were evaluated using 17 gait parameters selected from the literature. The variation of these parameters from HS values was combined to generate a multidimensional visual tool, referred to as a semiogram.

**Results:**

For both cohorts, all criteria showed moderate to very high test–retest reliability for intra-session measurements. Inter-session quantification was also moderate to highly reliable for all criteria except smoothness, which was not reliable for HS participants. All partial scores, except for the stability score, differed between the two populations. All partial scores were correlated with an objective but not subjective quantification of gait severity in the pMS population. A deficit in the pyramidal tract was associated with altered scores in all criteria, whereas deficits in cerebellar, sensitive, bulbar, and cognitive deficits were associated with decreased scores in only a subset of gait criteria.

**Conclusions:**

The proposed multidimensional gait quantification represents an innovative approach to monitoring gait disorders. It provides a reliable and informative biomarker for assessing the severity of gait impairments in individuals with pMS. Additionally, it holds the potential for discriminating between various underlying causes of gait alterations in pMS.

## 1. Introduction

Multiple sclerosis (MS) is a chronic demyelinating disease of the central nervous system that can cause a variety of symptoms, including spasticity, fatigue, loss of balance, sensory deficits, and weakness. These impairments often interfere with gait, with up to 75% of people with MS reporting difficulties walking at some point during the course of their disease (1). Many patients rank complaints such as “heavy legs,” sensations of having to “fight their own leg,” and “running out of energy” as the most debilitating (2). These impairments have a significant impact on the daily activities, social status, and overall quality of life of people with MS and their loved ones.

Day-to-day evaluation of gait disturbances in MS primarily relies on detailed patient interviews and visual observation of walking. The Kurtzke Expanded Disability Status Scale (EDSS), a 0- to 10-point scale, is the main reported outcome measure, determined by gait and functional system (FS) scores, including Pyramidal, Cerebellar, Brainstem, Sensory, Bowel, and Bladder, Visual, Cerebral or Mental, and Other factors. EDSS scores ≤ 4.0 are determined by FS scores alone, whereas scores 4.0–9.5 represent both gait abilities and FS scores. However, this scale is criticized for its insensitivity to early alterations and slight changes (3), as well as its high inter-rater variability (4). To complement clinician assessment, patient-reported outcomes such as the Multiple Sclerosis Walking Scale-12 (MSWS) can inform mobility limitations (5). The MSWS is a 12-item measure of the impact of MS on walking, rated on a scale from 1 to 5, and reported from 0 to 100. However, patient-reported outcomes are subjective and may be confounded by psychosocial factors, disease-related cognitive impairments, unblinding and “expectation bias,” or “response shift” in longitudinal evaluations (6).

Objective gait can be measured in various ways. Stopwatch-timed tests, such as the timed up-and-go test or the 25-foot walk test, are considered the best objective measure of walking disability for MS (5) and have long been used (7, 8). However, these tests have high intra-subject variability (9) solely on gait performance, without taking into account its quality status. From a semiological standpoint, the visual qualitative description of gait disorders has long been and still remains the primary tool for clinicians. However, in some progressive diseases such as multiple sclerosis, gait degradation may be quantified at an infra-clinical scale: visual evaluation may lack sensitivity for small changes and cannot reliably capture the progression from one consultation to another (10). To refine the analysis, laboratory-based measurements are powerful tools that can track gait disturbances early on in clinically isolated syndromes (11). However, these tools are cumbersome and expensive, and cannot be implemented within the time constraints of a routine clinical examination.

Inertial measurement units (IMUs) are small, lightweight wearable sensors that can be used to assess gait in MS both in routine clinical practice and at home for long-term physiological gait assessment (7, 12–19). They have also been found useful to detect early changes in MS with clinically isolated syndromes (13, 20), and parameters such as speed, step length, and step time are correlated with the severity of the disease (16). Longitudinal monitoring of gait and balance identifies changes in disease progression that are modifiable with physical rehabilitation (21). The use of IMUs to study the impact of spasticity on MS gait has received increased support with the development of related treatments (22–24). Nevertheless, other causes of gait deterioration (ataxia, sensitive deficit, vestibular deficit, and cognitive deficit) have been little investigated.

In this study, we present a simple visual tool computed from IMU signals, called a semiogram, which enables a qualitative evaluation of gait for the longitudinal monitoring of patients with progressive MS (pMS), characterized by gradual accrual of disability independent of relapses over time, and including primary and secondary progressive MS. The primary objective of this tool is to assist the clinician in quantifying the degradation or improvement of each semiotic criterion of gait activity from one consultation to another in a patient. This study details the construction of the semiogram, evaluates its reliability among a group of pMS patients and healthy control subjects (HS) measured at different times, and finally shows that this representation is consistent with disease severity and functional status scores, such as MSWS, EDSS, and EDSS FS values, in individuals with pMS.

## 2. Methods

### 2.1. Cohorts

Participants with pMS were consecutively recruited from the outpatient clinic of Percy Hospital (Clamart, France) between June 2018 and September 2018. HS participants were recruited from the hospital and research unit staff between June 2018 and September 2018. All participants were followed for 12 months. The inclusion criteria for the pMS cohort were an age of at least 18 years, a diagnosis of primary progressive or secondary progressive MS according to the 2010 International Panel criteria (25), the ability to walk two sets of 10 m forward and back with a U-turn, and no other condition than neurological one linked to pMS that affects gait. The only exclusion criterion was pregnancy. Inclusion criteria for the HS cohort were no report of falls in the past 5 years before inclusion [falls were defined as events that lead the standing or walking individual to a lower level on the ground unintentionally, without being externally pushed or pulled, regardless of whether an injury is sustained (26)], no disease that could affect walking and to be considered healthy after a clinical examination by medical doctors among investigators. All participants provided written informed consent before inclusion. The study protocol was conducted according to the Helsinki principles and was approved by Ethics Committee “Protection des Personnes Nord Ouest III” (ID RCB: 2017-A01538-45).

### 2.2. Gait measurement protocol and processing

#### 2.2.1. Protocol

**Figure 1:**
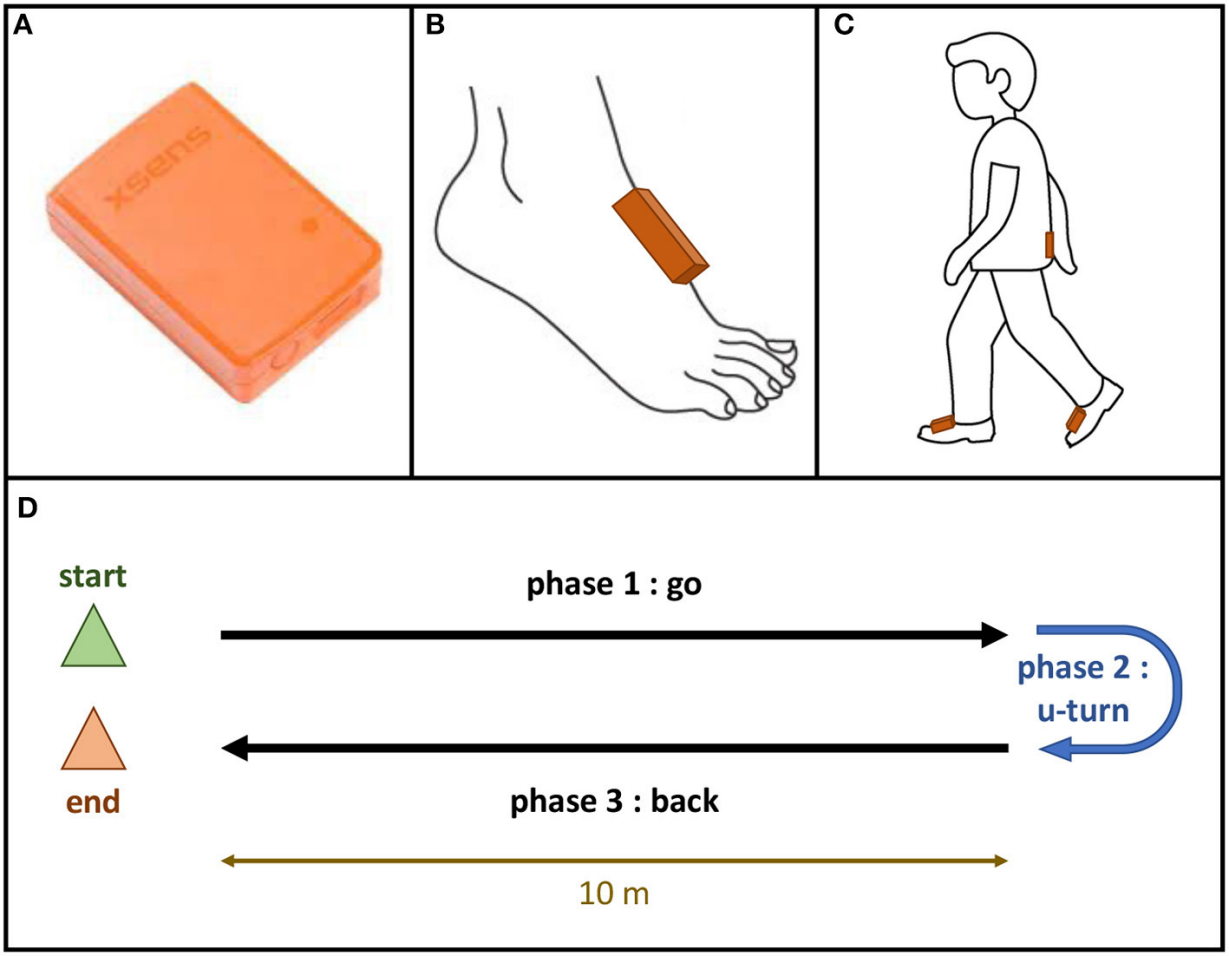
Gait protocol. **(A)** mTw Awinda XSens^®^ sensor; **(B)** Position of the sensor on the dorsal part of each foot; **(C)** Position of the sensors using Velcro bands: one sensor on each foot, one sensor in the lower back (vertebra L5); **(D)** Gait trial: 10-meter walk test with a U-turn.

Gait was measured by using three 3-D accelerometers (MTw Awinda XSens^®^, 100-Hz sampling frequency) positioned on the lower back (L4-L5 vertebrae) and dorsal part of both feet with elastic belts. All participants remained fully clothed and wore their own shoes, which remained consistent between all visits. Participants who used bracing or assistive devices for safety during gait used them during all walking trials. Each gait measurement included two 10-m walks with a U-turn and back, performed at a self-selected comfortable speed on an unlevelled floor. The start of each walking trial was verbally signaled by the assessor. The U-turn was to be performed behind a marked line on the ground, and both the strategy and side of the U-turn were left up to the patient's discretion. Patients were assessed three times at 6-month intervals. The protocol and sensor placement are shown in Figure 1.

#### 2.2.2. Gait detection

Gait detection was performed using an automated detection algorithm that was previously described (27). The algorithm combined pattern extraction and temporal dilation methods to detect gait events from the foot-level sensor data. Firstly, patterns corresponding to steps in multidimensional time series were extracted from foot-level sensors. Then, the algorithm accurately determined the two important walking events—Initial Contact (IC) and Final Contact (FC).

#### 2.2.3. U-turn detection

The U-turn was identified from the whole signal using a previously validated automated method (28). In summary, the method integrated the angular velocity around the craniocaudal axis derived from the IMU positioned on the lower back to extract the angular position around the craniocaudal axis. During the U-turn, the linear drift was corrected by assuming that the turn begins at 0° and ends at 180°. The method automatically detected inflection points close to these values and defined them as the boundaries of the U-turn. Overall, this automated method was able to accurately delineate the U-turn from the walking signal using IMU data.

### 2.3. Construction of the semiogram

#### 2.3.1. Inclusion and computation of potential parameters

The selection of parameters included in the multidimensional score was initially based on a systematic review of the use of inertial sensors in neurological populations, conducted by Vienne et al. (7), this selection was then complemented by more recent parameters validated in the literature (29–34). As recommended in this systematic review, walking speed was initially considered a global criterion for assessing gait quality. Then, the semiological analysis of walking was segmented into seven gait criteria that are commonly assessed in the fields of neurology, physical medicine, rehabilitation, gerontology, and rheumatology. The seven criteria were: springiness, smoothness, steadiness, sturdiness, stability, symmetry, and synchronization. To develop the multidimensional score, we selected the most relevant parameters for each of the seven gait criteria based on the systematic review, updated according to the literature. The calculation methods for each parameter were derived from the literature and are explained below. The use of consistent definitions and methods ensured the reliability and validity of the multidimensional score across different studies and populations.

- **Average speed:** refers to gait velocity.

− *Velocity (V)*. It was defined as the total length (20 m) divided by the total duration of the walking test (from the first to the last gait event) after the exclusion of the U-turn.

- **Springiness:** refers to gait rhythmicity. Two parameters were selected for inclusion:

− *Stride time (StrT)*. This allows for capturing springiness during straight walking. It was defined as the time between consecutive initial contact (IC) of the same foot, averaged across all strides within the trial after the initiation step, and excluding the U-turn period. This definition ensures that only valid strides are included in the calculation of stride time.
− *U-turn time (UtrT)*. This allows for capturing springiness during U-turn. It was defined as the duration of the turn that was segmented using the method described above.

- **Smoothness:** refers to gait continuousness or non-intermittency (35). Three parameters were selected for inclusion, as recommended by Melendez-Calderon et al. (29):

− *Spectral arc length (SPARC*−*G)*. It measures the smoothness of the trunk signal by analyzing its frequency components by calculating the arc length of the Fourier magnitude spectrum of the gyration signal within an adaptive frequency range. This index quantifies movement intermittencies independently of its amplitude or duration (29, 30, 32–34). The computation procedure was performed according to the method described by Melendez-Calderon et al. (29).
− *Log dimensionless jerk (LDLJ*−*A)*. This quantifies how quickly the acceleration of the signal is changing over time, taking into account amplitude and duration. We computed the anteroposterior jerk of the trunk sensor, during straight-walking phases as the variation of the anteroposterior acceleration (35). The computation procedure was the same as described in Melendez-Calderon et al. (29). A longer spectrum indicates a rougher signal.

- **Steadiness:** refers to gait regularity. Four parameters were selected for inclusion:

− *Variation coefficient of step time (CV*_*StrT*_*)*. This allows for capturing springiness variation along the test. It was defined as the standard deviation of the vector of stride times divided by its average.
− *Variation coefficient of double stance time (CV*_*dstT*_*)*. This allows for capturing synchronization variation along the test. It was defined as the standard deviation of the vector of double stance times divided by its average.
− *Craniocaudal step autocorrelation coefficient (P*1_*aCC*_*)*. It evaluates how similar the signal of the trunk is at a time delay corresponding to the duration of a step. This similarity is quantified by the first peak of the craniocaudal autocorrelation coefficient of the lower back.
− *Craniocaudal stride autocorrelation coefficient (P*2_*aCC*_*)*. It evaluates how similar the signal of the trunk is at a time delay corresponding to the duration of a stride. This similarity is quantified by the second peak of the craniocaudal autocorrelation coefficient of the lower back.

- **Sturdiness:** refers to gait amplitude. One parameter was selected for inclusion:

− *Step length (SteL)*. It is an indirect indicator of sturdiness that reflects the solidity and robustness of the gait. It was defined as the total length (20 m) divided by the total number of steps after the exclusion of the U-turn.

- **Stability:** refers to gait balance. One parameter was selected for inclusion:

− *Mediolateral root mean square (RMS*_*aML*_*)*. It quantifies the side-to-side movement of the trunk during the test. It was defined as the measure of the dispersion of the mediolateral acceleration of the lower back relative to zero during straight-walking phases.

- **Symmetry:** refers to right/left concordance during gait. Five parameters were selected for inclusion:

− *Ratio of the step to the stride peak of the craniocaudal correlation coefficient (P*1*P*2_*aCC*_*)*. This quantifies the symmetry of the resultant acceleration at the trunk level during left and right activities. It was defined as the ratio of P1 to P2, P1 and P2 previously defined.
− *Ratio of left and right mean swing times (swTr)*. This quantifies the symmetry of the left and right activities. It was defined as the ratio of the minimum (right or left) of averaged swing time divided by the maximum (right or left) of averaged swing time.
− *Three improved harmonic ratios: anteroposterior (iHR*_*aAP*_*), mediolateral (iHR*_*aML*_*), craniocaudal (iHR*_*aCC*_*)*. They evaluate the similarity of the energy distribution as a function of frequency between the left and right limbs. The use of the harmonic ratio to describe gait smoothness was first introduced by Gage (36) and later improved by Pasciuto et al. (37). The iHRs quantify the biphasic and monophasic natures of the signals, which are part of gait symmetry (38). The computation procedure was as described (31). To sum up, for each stride, a fast Fourier transform was performed to draw the Fourier series of the stride. The iHRs were calculated as the ratio of the power of the intrinsic harmonics (even harmonics along the anteroposterior and craniocaudal axes, odd harmonics along the mediolateral axes) to the total power of the signal. The result is a normalized index ranging from 0 to 100%.

- **Synchronization:** refers to inter-limb coordination during gait. One parameter was selected for inclusion:

− *Double stance time (dstT):* It assesses the synchronization between the lower limbs by quantifying the time during which both feet are in contact with the ground simultaneously. It was defined as the time between the IC of one foot and the FC of the contralateral foot divided by the total time of the cycle time. Two periods of double stance occur during a cycle and are therefore added before division by the total time of the cycle time.

#### 2.3.2. Selection of included parameters based on their reliability

For each parameter, we used a test-retest design to compute intra- and inter-session reliability. Intraclass correlation coefficients (ICCs) and standard error of the mean (SEM) values were calculated for all participants (both pMS and HS). To determine the level of agreement, we followed previous studies (39, 40). ICCs ≥0.75 were considered excellent, 0.4–0.75 were considered moderate to high, and ≤ 0.4 were considered low. Parameters with ICCs of 0.4 or lower were removed from the calculation of the gait criteria partial scores.

#### 2.3.3. Construction of the criteria partial scores

The z-score normalization method was used for each parameter in the study. This method allows for a comparison of the results across individuals and groups by transforming the raw data into a standardized score that reflects the distance from the mean in terms of standard deviation units. The z-score was computed for each participant based on the average value of the corresponding parameter in the reference group of HS in the same age range. A positive or negative z-score indicates that the value of the parameter for the participant is above or below the average value in the reference group, respectively. To facilitate interpretation, a z-coefficient of 1 or -1 was assigned to each parameter to indicate whether an increase in the parameter was considered beneficial or pathological, respectively. For gait criteria that included multiple parameters, the z-scores were merged with an arithmetic average to provide a single z-score for the criterion.

### 2.4. Clinical outcomes

The clinical outcomes assessed in pMS patients included both global severity outcomes, such as the Expanded Disability Status Scale (EDSS) and the Multiple Sclerosis Walking Scale-12 (MSWS), and functional severity scores, such as the Functional Scores (FS) obtained from the EDSS scale. Trained neurologists from Percy Hospital evaluated the FSs and total EDSS values before each trial. The MSWS was self-completed by the participants before each walking test visit.

### 2.5. Statistical analysis

#### 2.5.1. Reliability

A test retest design was chosen to evaluate the variability of the measurement between intra-session and inter-session evaluations for both HS and pMS participants. Relative reliability was computed by using two models of the ICC, ICC(1,1) and ICC(3,1), which both assess the relative reliability of single measurements (41). ICC(1,1) is based on the hypothesis that all within-subject variability is due to measurement error and ICC(3,1) assumes that this variability is caused by systematic bias different from measurement error. Heteroskedasticity, a property of a variable that shows non-constant standard deviations across observations, could cause misinterpretation of the ICC (42). Thus, heteroskedasticity was ruled out by testing the Pearson correlation coefficient (r) between the absolute differences and the individual mean values against the null hypothesis. A low ICC can be due to within-subject variability or narrow ranges of values within the sample (22). To distinguish between these two explanations, the SEM was computed as a measure of absolute reliability.

#### 2.5.2. Coherence

To ensure the relevance of each criterion, correlations between the parameters within each criterion and with other parameters were assessed. If there was a negative correlation between two parameters within a criterion, the less informative parameter was excluded from the criterion.

#### 2.5.3. Differences between groups

For each gait criterion and parameter, z-scores differences between groups were analyzed using the Mann-Whitney *U*-test.

#### 2.5.4. Correlations with gait severity and functional status

Correlation of z-scores with EDSS values and patient-reported outcomes as well as functional scores of the EDSS scale was assessed by the Pearson correlation coefficient and tested with the Fisher exact test.

Primary data analysis involved using MATLAB^®^ R2020b and Python 3.8 and statistical analysis involved R v3.5.3. All analyses used two-sided tests, and *p* ≤ 0.05 was considered statistically significant. Correction for multiple comparisons using Bonferroni adjustment was applied for all tests.

## 3. Results

### 3.1. Cohorts

Twenty individuals with pMS (10 females) and 19 age-matched HS (12 females) were enrolled in this longitudinal prospective study (Table 1). The mean age was 58 (SD 14) and 54 (SD 9), respectively. In the pMS cohort, the median EDSS was high (5.5 [quartile 1 (Q1)-Q3: 3.5 6.0]) and seven patients needed a walking aid to perform the test. Two participants from the HS cohort could not perform the measurement at 12 months. Therefore, only their first two visits were included. Four participants from the pMS cohort could not perform the measurement at 18 months and only their first three visits were included. Thus, we had 110 trials from HS participants and 168 trials from pMS patients.

**Table 1:** Baseline characteristics of individuals with progressive multiple sclerosis (pMS) and healthy subjects (HS).

**Characteristics**	**pMS (*n* = 22)**	HS (*n* = 19)^*^
Sex (M/F)	11/11	12/7
Age (years)	58 (14)	51 (17)
Height (m)	1.72 (0.08)	1.71 (0.06)
Weight (kg)	70.4 (15.3)	71.7 (14.3)
Body mass index (kg/m^2^)	23.6 (4.1)	24.3 (4.3)
**Concerning pMS**		-
Years since diagnosis	16 (10)	-
- *Years since first sign*	22 (15)	-
- *Years since progression*	11 (10)	-
Expanded Disease Status Scale	5.5 [3.5–6]	-
- *Pyramidal function*	3 [3–3]	-
- *Cerebellar function*	2 [0–3]	-
- *Bulbar function*	0 [0–1]	-
- *Sensitive function*	2 [0.5–2]	-
- *Cognitive function*	1 [0–2]	-
Multiple Sclerosis Walking Scale	65.72 (18.60)	-
Fatigue Impact Scale	42.74 (22.15)	-
Walking aid for the test (yes/no)	7/15	-
- *Cane (1 or 2)*	6	-
- *Walker*	1	-
- *Human help*	0	-
- *Cane and human help*	0	-

Mean (SD) or median [Q1–Q3] are given. ^*^Data are missing for 2 HS subjects.

### 3.2. Construction and validity of the semiogram

#### 3.2.1. Selection of reliable parameters

ICC(1,1) and ICC(3,1) values and SEMs are reported in Table 2 for each of the 17 parameters considered for inclusion.

**Table 2:** Reliability of parameters tested for inclusion in the semiogram.

		**Intra-session**			**Inter-session**		
**Criteria**	**Parameter**	**ICC(1,1)**	**ICC(3,1)**	**SEM**	**ICC(1,1)**	**ICC(3,1)**	**SEM**
Average speed	V (m/s)	0.97	0.97	0.01	0.96	0.96	0.01
Springiness	StrT (s)	0.98	0.98	0.01	0.93	0.93	0.02
	UtrT (s)	0.91	0.91	0.07	0.81	0.81	0.14
Smoothness	LDLJ-A (-)	0.91	0.91	0.02	0.82	0.82	0.04
	SPARC-G (-)	0.88	0.88	0.08	0.83	0.83	0.12
Steadiness	CVStrT (%)	0.78	0.78	0.12	0.82	0.82	0.15
	CVdstT (%)	0.49	0.49	0.15	0.64	0.64	0.21
	P1aCC (-)	0.94	0.94	0.01	0.90	0.90	0.01
	P2aCC (-)	0.88	0.88	0.01	0.87	0.87	0.01
Sturdiness	SteL (m)	0.96	0.96	0.00	0.95	0.95	0.01
Stability	RMS_*aML*_ (m/s^2^)	0.95	0.95	0.01	0.90	0.90	0.02
Symmetry	iHR_*aAP*_ (%)	0.97	0.97	0.25	0.93	0.93	0.53
	iHR_*aML*_ (%)	0.95	0.95	0.22	0.86	0.86	0.46
	iHR_*aCC*_ (%)	0.97	0.97	0.20	0.90	0.90	0.59
	P1P2_*aCC*_ (-)	0.56	0.56	0.01	0.53	0.53	0.02
	swT_*r*_ (-)	0.71	0.71	0.00	0.72	0.72	0.00
Synchronization	dstT (%)	0.96	0.96	0.19	0.94	0.94	0.38

Intraclass correlation coefficients [ICC(1,1) and ICC(3,1)] provide measures of relative reliability and standard error of the mean (SEM) values provide measures of absolute reliability.

**Figure 2:**
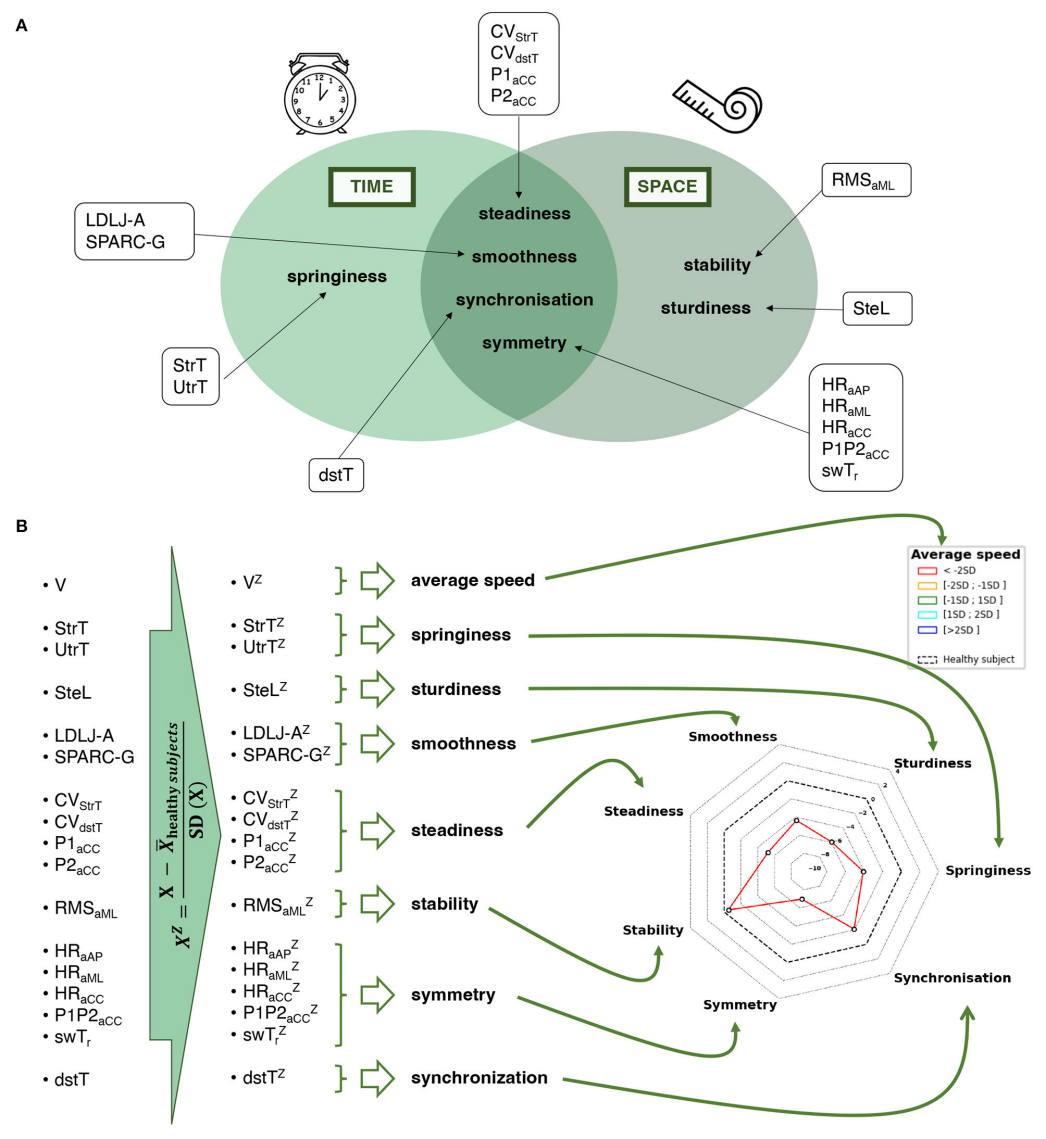
Description of a semiogram. **(A)** Criteria and parameters that make up the semiogram. **(B)** Computation of the semiogram after the recording of gait signals. V, velocity; SteL, step length; StrT, stride time; UtrT, U-turn time; LDLJ-A, log-dimensionless jerk computed from the trunk acceleration; SPARC-G, spectral arc length computed from the trunk gyration; CVStrT, coefficient of variation of the stride time; CVdstT, coefficient of variation of the double stance time; P1aCC, step autocorrelation coefficient of the trunk craniocaudal acceleration; P2aCC, stride autocorrelation coefficient of the trunk craniocaudal acceleration; RMSaML, root mean square of the trunk mediolateral acceleration; iHRaAP, improved harmonic ratio of the trunk anteroposterior acceleration; iHRaML, improved harmonic ratio of the trunk mediolateral acceleration; iHRaCC, improved harmonic ratio of the trunk craniocaudal acceleration; P1P2aCC, ratio P1 to P2; swTr, ratio of left and right swing times; dsT, double stance time.

All parameters showed moderate to very high agreement (ICC ≥ 0.4) and were therefore included in the final score for the semiogram. Included parameters and corresponding criteria are illustrated in Figure 2A.

#### 3.2.2. Normative data from the reference group

For each of the 17 selected qualitative parameters, the mean and SD were computed for the reference group of 19 HS participants by using the whole set of trials (two trials for each participant at each visit, i.e., six trials per HS participant). The results are displayed in Table 3.

**Table 3:** Mean, SD, and z-coefficient for included gait features for the reference group of 19 HS (six trials per participant, except for two participants without data at 12 months), for a total of 110 trials.

**Criteria**	**Parameter**	**Mean**	**SD**	***Z*-coefficient**
Average speed	V (m/s)	1.22	0.20	+
Springiness	StrT (s)	1.10	0.09	-
	UtrT (s)	2.62	0.75	-
Smoothness	LDLJ-A (-)	–8.07	0.35	+
	SPARC-G (-)	–5.37	0.84	-
Steadiness	CVStrT (%)	2.34	0.97	-
	CVdstT (%)	5.63	2.07	-
	P1aCC (-)	0.82	0.10	+
	P2aCC (-)	0.82	0.10	+
Sturdiness	SteL (m)	0.68	0.08	+
Stability	RMS_*aML*_ (m/s^2^)	1.28	0.33	-
Symmetry	iHR_*aAP*_ (%)	95.48	2.13	+
	iHR_*aCC*_ (%)	94.88	3.10	+
	iHR_*aML*_ (%)	86.77	6.32	+
	P1P2_*aCC*_ (-)	0.96	0.04	+
	swT_*r*_ (-)	0.96	0.03	+
Synchronization	dstT (%)	23.34	3.50	-

For the z-coefficient, + and - respectively corresponds to 1 or - 1, and indicate whether an increase in the parameter is considered beneficial or pathological.

#### 3.2.3. Construction of the semiogram

**Figure 3:**
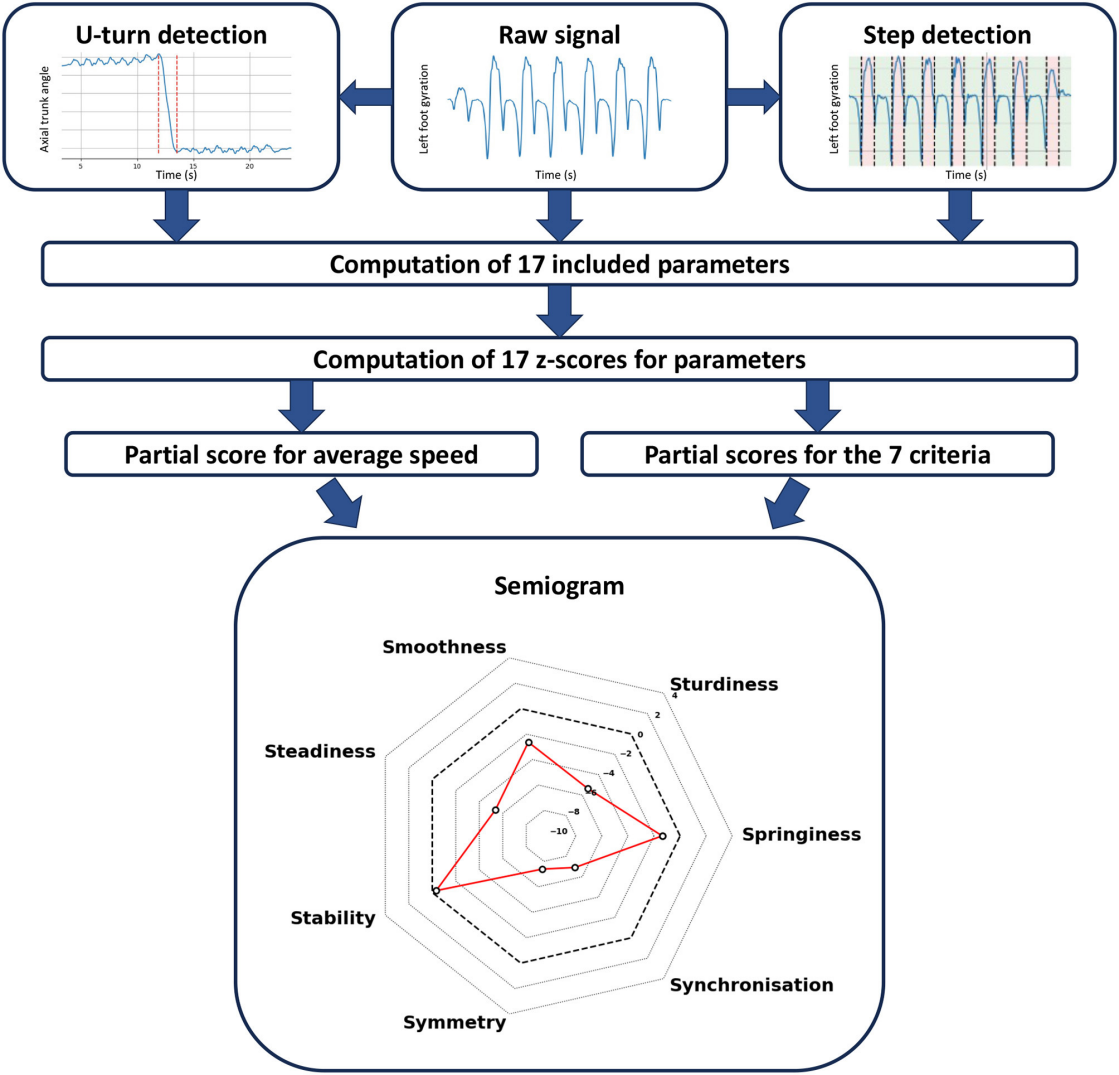
Method that drove the conception of the semiogram. The featured semiogram is an example of an individual with altered gait along several criteria (EDSS = 6).

The process for constructing the semiogram given the included parameters is outlined in Figure 2B. All stages of the construction, from the signal to the final radar plot, are schematized in Figure 3.

### 3.3. Validity of the semiogram

#### 3.3.1. Intra-session test-retest of criteria scores for HS and pMS participants

**Figure 4:**
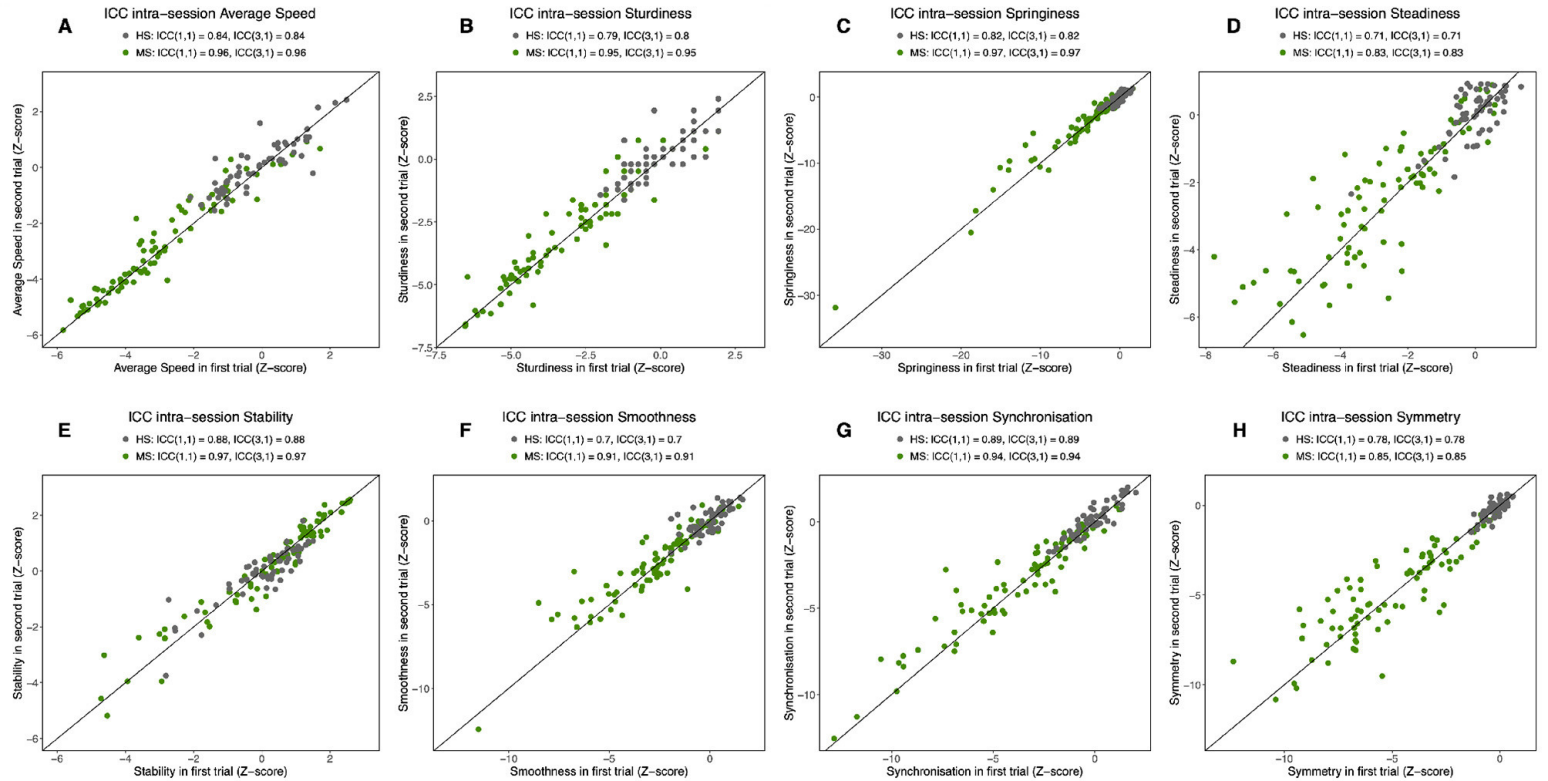
Intra-session intraclass correlation coefficients (ICCs). For speed **(A)** and the seven criteria for the semiogram: sturdiness **(B)**, springiness **(C)**, steadiness **(D)**, stability **(E)**, smoothness **(F)**, synchronization **(G)**, and symmetry **(H)**. ICCs are reported for the two populations separately: progressive multiple sclerosis (pMS) and healthy subjects (HS). ICC(1,1) supposes that subject variability is due to measurement error.

Measures of intra-session relative reliability are plotted in Figure 4, with distinct ICCs computed for HS and pMS participants. In the HS group, we observed excellent degrees of the intra-session agreement for speed and all qualitative criteria except steadiness and smoothness, which were moderate. A low SEM (steadiness: 0.06 z-score; smoothness: 0.05 z-score) indicates that the small range might be partly causing the low ICC. In the pMS group, speed and all qualitative criteria showed very high test–retest relative reliability, without exception. The detailed results for each parameter are provided in Supplementary material 1.

#### 3.3.2. Inter-session test–retest for HS and pMS participants

Measures of inter-session relative reliability between M0 and M6 were also computed for criteria (Supplementary material 2), with distinct ICCs computed for HS and pMS participants. In the HS group, ICCs were excellent or moderate, except one low but close to 0.4 for smoothness. All criteria showed very high reliability for the pMS group. The detailed results for each parameter are provided in Supplementary material 3.

#### 3.3.3. Correlation between the parameters

Within each criterion, the correlations between parameters were always >0.3. No parameters were excluded at this validation stage.

### 3.4. Difference between pMS and HS

**Figure 5:**
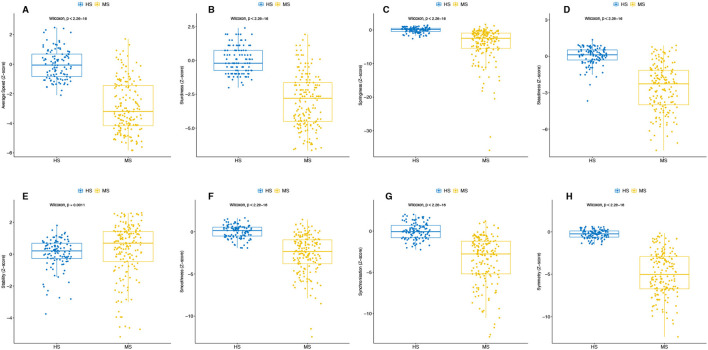
Difference in speed and each criterion of the semiogram between the two populations (HS and pMS). For speed **(A)** and the seven criteria for the semiogram: sturdiness **(B)**, springiness **(C)**, steadiness **(D)**, stability **(E)**, smoothness **(F)**, synchronization **(G)**, and symmetry **(H)**. All tests of the participants are included in the analysis. The *p*-value from the Wilcoxon test is reported.

When comparing pMS and HS participants, speed and all criteria but stability significantly differed (*p* ≤ 0.0001). Results are displayed in Figure 5. The detailed results for each parameter are provided in Supplementary material 4.

### 3.5. Use for longitudinal follow-up of pMS

#### 3.5.1. Correlation with disease global severity (EDSS, MSWS)

All the semiogram criteria were highly correlated to the validated general severity EDSS score. The subjective questionnaire score (MSWS) was moderately correlated with speed, springiness, smoothness, and steadiness, and was independent of the four other criteria (Table 4).

**Table 4:** Correlation coefficients for the semiogram criteria with the EDSS and MSWS scores.

	**MSWS**		**EDSS**	
	**r**	***p-value***	**r**	***p-value***
Speed	–0.18	**0.024**	–0.74	**≤0.001**
Springiness	–0.20	**0.012**	–0.50	**≤0.001**
Smoothness	–0.24	**0.003**	–0.63	**≤0.001**
Steadiness	–0.18	**0.026**	–0.59	**≤0.001**
Sturdiness	–0.15	0.062	–0.70	**≤0.001**
Stability	0.00	0.987	0.33	**≤0.001**
Symmetry	0.03	0.727	–0.44	**≤0.001**
Synchro	–0.05	0.543	–0.63	**≤0.001**

Significant correlations appear in bold type.

**Figure 6:**
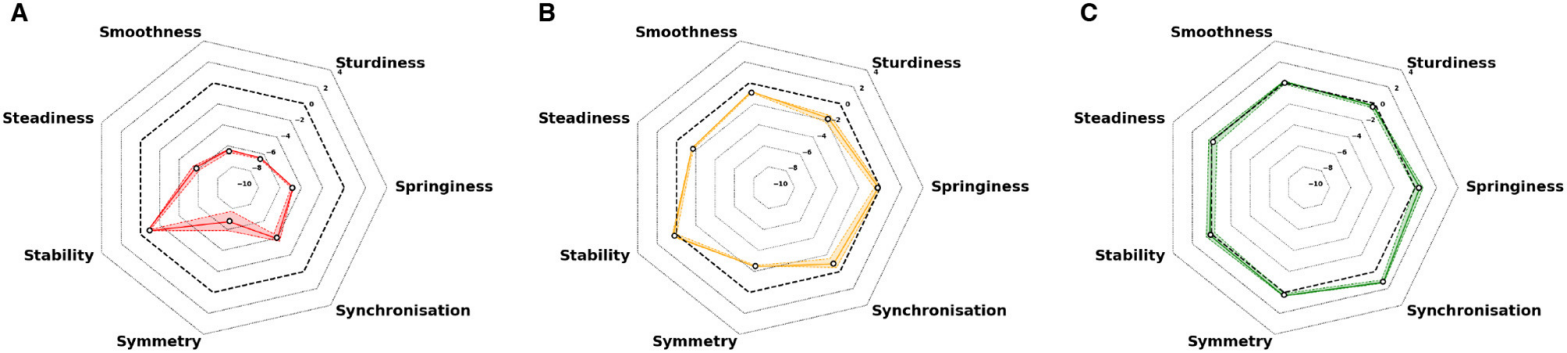
Representative examples of semiograms for two trials for three individuals. **(A)** pMS patient with severe disease (EDSS 6.0). **(B)** pMS patient with less severe disease (EDSS 3.5). **(C)** Healthy subject. The dashed black line represents the normative values (0 EDSS scores). Colored plain lines represent the arithmetic average of the two tests, and colored areas represent the extreme values of the session.

Examples of a semiogram for an individual with severe disease (EDSS 6.0) and less severe disease (EDSS 3.5) and an HS are given in Figure 6.

#### 3.5.2. A window into the functional status of the disease: correlation with functional status scores

All criteria were moderately to strongly associated with the Pyramidal FS (positive association for stability and negative for the others; Table 5). Cerebellar alterations were also positively correlated with stability and negatively correlated with all other criteria except symmetry. Sensitive lesions were associated with altered symmetry and improved stability. The bulbar sub-score was associated with altered speed, synchronization, and sturdiness. The cognitive sub-score was associated with altered speed, smoothness, steadiness, and sturdiness.

**Table 5:** Correlation coefficients for the semiogram criteria with the functional sub-scores.

	**EDSS**									
	**Pyramidal**		**Cerebellar**		**Sensitive**		**Bulbar**		**Cognitive**	
	**r**	***p-value***	**r**	***p-value***	**r**	***p-value***	**r**	***p-value***	**r**	***p-value***
Speed	–0.64	**≤0.001**	–0.52	**≤0.001**	–0.11	0.182	–0.24	**0.006**	–0.33	**≤0.001**
Springiness	–0.52	**≤0.001**	–0.16	0.068	–0.10	0.241	–0.09	0.307	–0.10	0.229
Smoothness	–0.52	**≤0.001**	–0.33	**0.001**	–0.05	0.597	–0.09	0.312	–0.18	**0.038**
Steadiness	–0.39	**≤0.001**	–0.37	**≤0.001**	0.10	0.233	–0.07	0.451	–0.40	**≤0.001**
Sturdiness	–0.56	**≤0.001**	–0.53	**≤0.001**	–0.04	0.657	–0.23	**0.007**	–0.41	**≤0.001**
Stability	0.44	**≤0.001**	0.34	**≤0.001**	0.31	**≤0.001**	0.13	0.139	–0.01	0.907
Symmetry	–0.24	**0.004**	–0.04	0.670	0.23	**0.005**	–0.08	0.363	–0.05	0.589
Synchro	–0.64	**≤0.001**	–0.27	**0.001**	–0.13	0.136	–0.21	**0.015**	0.01	0.914

Significant correlations appear in bold type.

#### 3.5.3. An example of longitudinal follow-up

**Figure 7:**
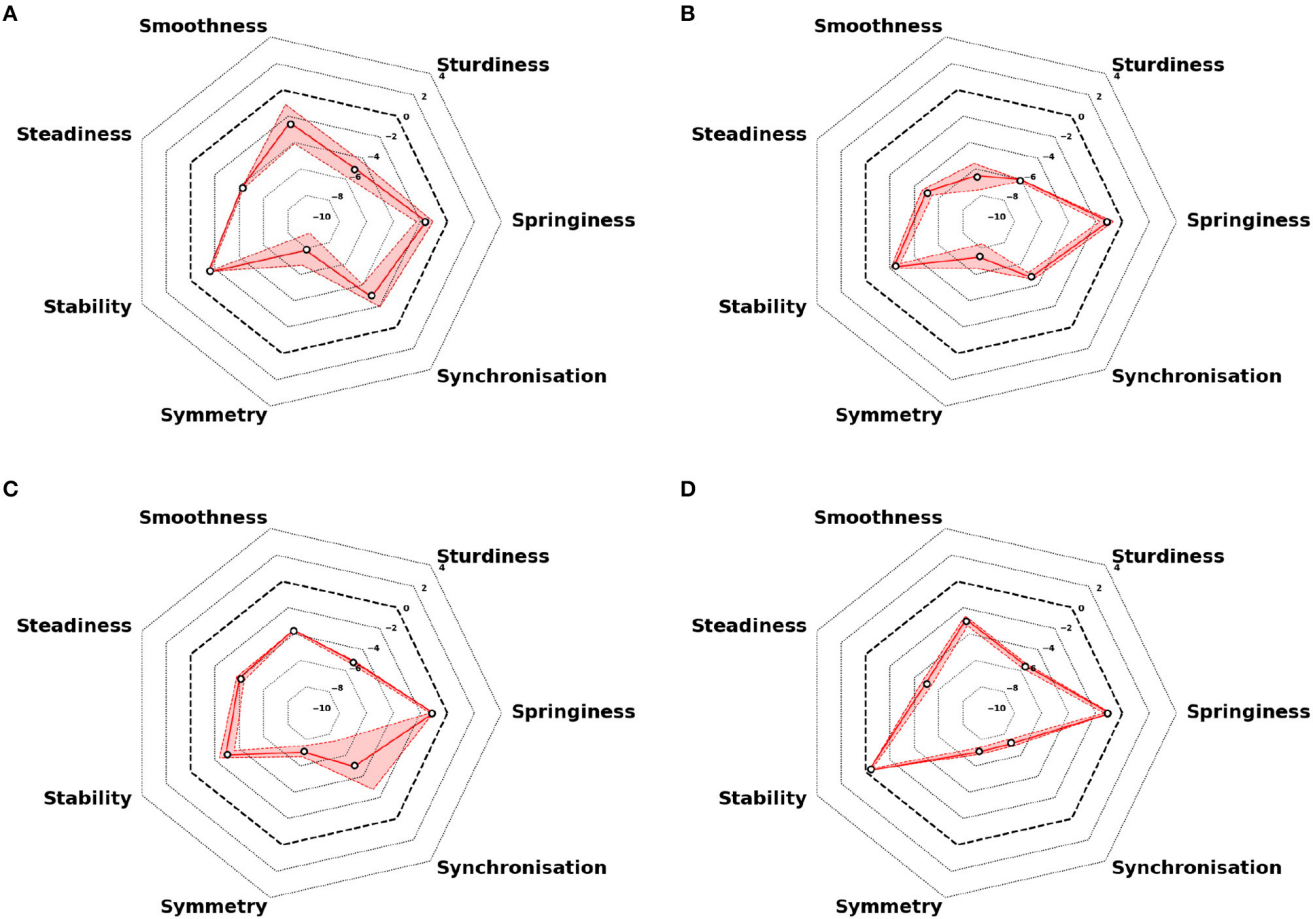
Repeated semiograms for follow-up over an 18-month period for an individual with high disability. **(A)** Measures at inclusion (M0): EDSS 6.0. **(B)** Measures after 6 months (M6): EDSS 6.0. **(C)** Measures after 12 months (M12): EDSS 6.5. **(D)** Measures after 18 months (M18): EDSS 6.5. The dashed black line represents the normative values (0 EDSS scores). Colored plain lines represent the arithmetic average of the two tests, and colored areas represent the extreme values of the session.

The longitudinal follow-up over an 18-month period is illustrated in Figure 7 for an individual with a high disability. The EDSS at inclusion was 6.0 and remained at that level at month 6. It then increased to 6.5 at month 12. At month 18, the EDSS was again 6.5. A qualitative analysis of semiograms at different times shows that the individual displayed higher differences than the norm at months 12 and 18 (Figures 7C, D) as compared with months 0 and 6 (Figures 7A, B).

## 4. Discussion

In the present study, we introduce the semiogram, a novel multidimensional approach for automated assessment and visualization of gait in routine neurological practice. The main objective of this concentric sector chart is to allow physicians to quantitatively assess the semiological characteristics of their patients' gait in relation to the general population and to track the evolution of each criterion throughout the pathology. Each branch of the chart represents a semiological criterion of the patient's gait, such as springiness, smoothness, steadiness, sturdiness, stability, symmetry, or synchronization. Average speed, which is recognized in the literature as a global gait parameter (7), influences the resulting color of the chart.

The semiogram is suitable for clinical practice. It is based on the use of three easily deployable IMUs in a short-distance test and does not require any specific space or platform. Even if using a short walking test may reduce the robustness of some parameters, they are commonly used in clinical practice as they are more practical and easier to conduct (7), particularly for individuals with severe pMS who may find longer walking tests difficult to complete. It has also been found that uninterrupted walking for more than 2 min is not common in real-life situations (43). Moreover, a correlation exists between the gait assessed in the laboratory and the daily ambulation (17). Most parameters of the semiogram require a walking event segmentation algorithm. To obtain a tool deployable in routine clinical practice, this segmentation should be automated without manual review, which remains a challenge in gait quantification. Detecting highly degraded steps, such as those in our cohort, is often difficult with conventional algorithms and requires a previously validated algorithm (27). Finally, the visual interpretation of the semiogram and the ability to overlay examinations from multiple months apart provide valuable assistance to clinicians in the quantitative analysis of gait data.

The semiogram is a reproducible tool, consistent with the semiological and clinical descriptions of gait in the literature. Indeed, the originality of our study lies in the selection of potential parameters based on a systematic literature review and their clinical application in patients with pMS (7), which enhances the external validity of our approach. Even if the initial literature review only included 78 studies due to quality selection issues (7), we updated it with a permanent bibliographic survey. We then included the parameters more recently appeared, in particular in the case of smoothness for which there was a consequent number of publications (29). For both HS and pMS cohorts, the intra-session test-retest agreement is moderate to very high for all included parameters and criteria (Table 2), which demonstrates the reproducibility of the measurements under the conditions of the 10-m walking test. Inter-session test-retest agreement is also moderate to very high comparing M0 and M6 (Table 2), thus validating the use of the semiogram for bi-annual visits, which is a frequent practice in neurological care. Previous research has also investigated other multidimensional gait scores in a free-living environment (44, 45) or in a clinical environment, such as the study of Mansour et al., who developed a multifeatured gait score and evaluated it in an older population (40). Several differences from this score enhance the relevance of our multidimensional score. In our study, the gait criteria are based on clinical standards (46) and therefore follow closely the clinical evaluation currently done in routine practice. Unlike the multi-feature gait score proposed by Mansour et al., we evaluate the reliability of each parameter separately before inclusion and do not eliminate redundancy in our semiogram. Beyond the difference in method regarding the selection of parameters and their categorization into different criteria, it is important to note that the internal validity, assessed through the reproducibility of measurements, is similar in both studies, based on similar walking tests.

The semiogram is clinically relevant. On a macroscopic level, it differentiates indeed pathological participants from healthy subjects. The evaluation of parameters and criteria consistently shows a significant distinction between the two cohorts in all instances, except for stability. Specifically, pMS individuals exhibited shorter steps (lower sturdiness), lower cadence and higher U-turn time (lower springiness), higher variability (lower steadiness), higher perturbations within their walk (lower smoothness), higher double stance time (lower synchronization between right and left cycle), and more asymmetrical behavior (lower symmetry) than HS individuals. At a more granular level, the semiogram off ers valuable insights into disease severity and functional status of patients. In terms of disease global severity scores, the objective EDSS test exhibits a highly significant negative correlation with all criteria of the semiogram, except for stability. This finding aligns with the majority of studies and meta-analyses that have explored parameters such as velocity, step length, and average step duration, which are widely investigated in the field (16, 47, 48). These observations are also in line with the results obtained with more global approaches used to analyze the signal obtained with IMUs (49). Other parameters that necessitate precise segmentation of walking events have received less attention in research. Concerning stability, it is observed to increase with disease severity. This phenomenon has been previously attributed to decreased walking speed in severe diseases, an increase of double stance time, and a reduction in swing phase amplitude (50). In our study, only one parameter is used for stability assessment, the mediolateral root mean square. Recent studies have described other parameters that accurately reflect stability in neurological pathologies, such as the local divergence exponent (51). This parameter could be investigated to confirm if the trend holds true. According to the existing literature, the MSWS, which provides a subjective assessment of the patient's disease activity, seems to exhibit a weaker correlation with gait deterioration than EDSS (16). However, some studies emphasize that because being based on the patient's perception, the MSWS test may actually be more effective than objective scores in detecting significant gait deterioration (52). However, due to the limited sample size, we are unable to examine whether the semiogram can effectively monitor changes over time. The statistical power is insufficient, as only four out of 22 participants with pMS experienced a 1-point or greater change in their EDSS status during the follow-up period. To overcome this limitation, future studies should involve a larger sample size and a longer follow-up duration to provide more robust findings. Regarding functional status, the results confirm that pyramidal and cerebellar disorders are the primary contributors to gait degradation in the pMS cohort (11). Furthermore, various gait features have been identified as capable of distinguishing between individuals with pMS who exhibit predominant pyramidal dysfunctions and those with predominant cerebellar dysfunctions. For instance, individuals with pyramidal dysfunctions demonstrated significantly lower cadence, longer double stance time, and more asymmetrical gait compared to those with cerebellar dysfunctions (53). The semiogram enables the decomposition of gait analysis and facilitates the identification of associations between criteria and functional impairments. Our study revealed that reduced springiness and symmetry were specifically associated with pyramidal dysfunction rather than cerebellar tract disorders, which aligns with previous research findings (11). These findings highlight the importance of considering the specific type of dysfunction when assessing gait in pMS patients.

Another advantage of the semiogram relies on its capacity to integrate multiple validated parameters, enabling a comprehensive and sensitive analysis of gait. This approach ensures that each criterion is captured as accurately as possible. In our study, the construction of the semiogram is based on parameters commonly utilized in neurological disorders, rather than being specific to pMS (29, 30, 34, 54). Therefore, the applicability of this method can be readily extended to other neurological disorders by conducting a subsequent literature search. This approach eliminates the limitations associated with relying solely on a single parameter and allows for a more comprehensive assessment of gait across various neurological conditions (55). Another concern is the resolution of the semiogram. We have used one standard deviation (SD). However, it should be noted that the z-scores of the various parameters represented can be decimal numbers. Furthermore, Bohannon et al. have indicated that even small changes in gait parameters can have clinical significance (56). Interpretation of the semiogram and its correlation with the clinic is therefore variable, and depends in particular on the alteration in gait and the pathology being studied. It can therefore only be conceived in conjunction with the examination of the patient, and the resolution could be adapted to different patient cohorts. Indeed, further validation in other patient cohorts such as those with Parkinson's disease, Alzheimer's disease, cerebellar ataxia, and stroke, as reviewed by Vienne et al. (7), would be valuable. By applying the semiogram method to these different neurological conditions, we may assess its effectiveness and generalizability, expanding its utility beyond the specific context of progressive multiple sclerosis.

## 5. Conclusion

The wearable technology discussed in this study offers an innovative way to collect and visualize objective and quantitative data on mobility that was not previously available in a clinical setting. This technology has the potential to serve as an important outcome measure for evaluating mobility impairment in individuals with pMS. Furthermore, the use of evidence-based criteria and the semiogram visualization provides a quick and comprehensive assessment of gait that can be incorporated into clinical practice. The radar plot visualization may also aid both patients and clinicians in tracking disease progression over time.

## Data availability statement

The healthy subjects dataset and the online demonstration of the semiogram are accessible at the following address: https://www.ipol.im/.

## Ethics statement

The studies involving humans were approved by Protection des Personnes Nord Ouest III (ID RCB: 2017-A01538-45). The studies were conducted in accordance with the local legislation and institutional requirements. The participants provided their written informed consent to participate in this study.

## Author contributions

CV and NE conceived the study, participated in the analysis, and wrote the manuscript. AV-J, AM, and FQ conceived the study and participated in the data acquisition. FB, MS, M-LB, IT, CT, and ED participated in the data acquisition. DR conceived the study, participated in the data acquisition, and edited the manuscript. LO conceived the study and edited the manuscript. All authors contributed to the refinement of the study protocol and approved the final manuscript.

## References

[1] Bethoux F Bennett S. Evaluating walking in patients with multiple sclerosis: which assessment tools are useful in clinical practice? Int J MS Care. (2011) 13:4–14. 10.7224/1537-2073-13.1.4 24453700 PMC3882949

[2] Heesen C Böhm J Reich C Kasper J Goebel M Gold SM. Patient perception of bodily functions in multiple sclerosis: gait and visual function are the most valuable. Mult Scler. (2008) 14:988–91. 10.1177/1352458508088916 18505775

[3] Meyer-Moock S Feng YS Maeurer M Dippel FW Kohlmann T. Systematic literature review and validity evaluation of the Expanded Disability Status Scale (EDSS) and the Multiple Sclerosis Functional Composite (MSFC) in patients with multiple sclerosis. BMC Neurol. (2014) 14:58. 10.1186/1471-2377-14-58 24666846 PMC3986942

[4] Noseworthy JH Vandervoort MK Wong CJ Ebers GC. Interrater variability with the Expanded Disability Status Scale (EDSS) and Functional Systems (FS) in a multiple sclerosis clinical trial. The Canadian Cooperation MS Study Group. Neurology. (1990) 40:971–5. 2189084 10.1212/wnl.40.6.971

[5] Kieseier BC Pozzilli C. Assessing walking disability in multiple sclerosis. Mult Scler. (2012) 18:914–24. 10.1177/1352458512444498 22740603

[6] Kyte DG Draper H Ives J Liles C Gheorghe A Calvert M. Patient reported outcomes (PROs) in clinical trials: is “in-trial” guidance lacking? A systematic review. PLoS ONE. (2013) 8:60684. 10.1371/journal.pone.0060684 23560103 PMC3613381

[7] Vienne A Barrois RP Buffat S Ricard D Vidal PP. Inertial sensors to assess gait quality in patients with neurological disorders: a systematic review of technical and analytical challenges. Front Psychol. (2017) 8:1–12. 10.3389/fpsyg.2017.00817 28572784 PMC5435996

[8] Vienne-Jumeau A Quijoux F Vidal PP Ricard D. Value of gait analysis for measuring disease severity using inertial sensors in patients with multiple sclerosis: protocol for a systematic review and meta-analysis. Systemat Rev. (2019) 8:1–5. 10.1186/s13643-018-0918-z 30621765 PMC6325868

[9] Kragt JJ Van Der Linden F Nielsen JM Uitdehaag BMJ Polman CH. Clinical impact of 20% worsening on Timed 25-foot Walk and 9-hole Peg Test in multiple sclerosis. Mult Scler. (2006) 12:594–8. 10.1177/1352458506070768 17086905

[10] Martin CL Phillips BA Kilpatrick TJ Butzkueven H Tubridy N McDonald E. Gait and balance impairment in early multiple sclerosis in the absence of clinical disability. Mult Scler. (2006) 12:620–8. 10.1177/1352458506070658 17086909

[11] Kalron A Givon U. Gait characteristics according to pyramidal, sensory and cerebellar EDSS subcategories in people with multiple sclerosis. J Neurol. (2016) 16:6. 10.1007/s00415-016-8200-6 27314963

[12] Motta C Palermo E Studer V Germanotta M Germani G Centonze D. Disability and fatigue can be objectively measured in multiple sclerosis. PLoS ONE. (2016) 11:e148997. 10.1371/journal.pone.0148997 26863109 PMC4749243

[13] Pau M Mandaresu S Pilloni G Porta M Coghe G Marrosu MG. Smoothness of gait detects early alterations of walking in persons with multiple sclerosis without disability. Gait Post. (2017) 58:307–9. 10.1016/j.gaitpost.2017.08.023 28858779

[14] Pau M Corona F Pilloni G Porta M Coghe G Cocco E. Texting while walking differently alters gait patterns in people with multiple sclerosis and healthy individuals. Mult Scler Relat Disord. (2018) 19:129–33. 10.1016/j.msard.2017.11.021 29216541

[15] Pau M Caggiari S Mura A Corona F Leban B Coghe G. Clinical assessment of gait in individuals with multiple sclerosis using wearable inertial sensors: comparison with patient-based measure. Mult Scler Relat Disord. (2016) 10:187–91. 10.1016/j.msard.2016.10.007 27919488

[16] Vienne-Jumeau A Quijoux F Vidal PP Ricard D. Wearable inertial sensors provide reliable biomarkers of disease severity in multiple sclerosis: a systematic review and meta-analysis. Ann Phys Rehabil Med. (2020) 63:138–47. 10.1016/j.rehab.2019.07.004 31421274

[17] Shema-Shiratzky S Hillel I Mirelman A Regev K Hsieh KL Karni A. A wearable sensor identifies alterations in community ambulation in multiple sclerosis: contributors to real-world gait quality and physical activity. J Neurol. (2020) 267:1912–21. 10.1007/s00415-020-09759-7 32166481

[18] Ibrahim AA Küderle A Gaßner H Klucken J Eskofier BM Kluge F. Inertial sensor-based gait parameters reflect patient-reported fatigue in multiple sclerosis. J Neuroeng Rehabil. (2020) 17:9. 10.1186/s12984-020-00798-9 33339530 PMC7749504

[19] Zahn A Koch V Schreff L Oschmann P Winkler J Ganer H. Validity of an inertial sensor-based system for the assessment of spatio-temporal parameters in people with multiple sclerosis. Front Neurol. (2023) 14:1164001. 10.3389/fneur.2023.1164001 37153677 PMC10157085

[20] Müller R Hamacher D Hansen S Oschmann P Keune PM. Wearable inertial sensors are highly sensitive in the detection of gait disturbances and fatigue at early stages of multiple sclerosis. BMC Neurol. (2021) 21:337. 10.1186/s12883-021-02361-y 34481481 PMC8418019

[21] Fritz NE Newsome SD Eloyan A Marasigan RER Calabresi PA Zackowski KM. Longitudinal relationships among posturography and gait measures in multiple sclerosis. Neurology. (2015) 84:2048–56. 10.1212/WNL.0000000000001580 25878185 PMC4442106

[22] Balantrapu S Sosnoff JJ Pula JH Sandroff BM Motl RW. Leg spasticity and ambulation in multiple sclerosis. Mult Scler Int. (2014) 2014:1–7. 10.1155/2014/649390 24999434 PMC4066854

[23] Pau M Coghe G Corona F Marrosu MG Cocco E. Effect of spasticity on kinematics of gait and muscular activation in people with multiple sclerosis. J Neurol Sci. (2015) 358:339–44. 10.1016/j.jns.2015.09.352 26409825

[24] Ballester BR Antenucci F Maier M Coolen ACC Verschure PFMJ. Estimating upper-extremity function from kinematics in stroke patients following goal-oriented computer-based training. J NeuroEng Rehabil. (2021) 18:1–17. 10.1186/s12984-021-00971-8 34972526 PMC8720223

[25] Polman CH Reingold SC Banwell B Clanet M Cohen JA Filippi M. Diagnostic criteria for multiple sclerosis: 2010 revisions to the McDonald criteria. Ann Neurol. (2011) 69:292–302. 10.1002/ana.22366 21387374 PMC3084507

[26] Zecevic AA Salmoni AW Speechley M Vandervoort AA. Defining a fall and reasons for falling: comparisons among the views of seniors, health care providers, and the research literature. Gerontologist. (2006) 46:367–76. 10.1093/geront/46.3.367 16731875

[27] Voisard C De l'Escalopier N Ricard D Oudre L. Automatic gait events detection with inertial measurement units: healthy subjects and moderate to severe impaired patients. Res. Square [Preprint]. (2023). 10.21203/rs.3.rs-2792379/v1 PMC11184826 38890696

[28] Barrois RPM Ricard D Oudre L Tlili L Provost C Vienne A. Observational study of 180° turning strategies using inertial measurement units and fall risk in poststroke hemiparetic patients. Front Neurol. (2017) 8:1–11. 10.3389/fneur.2017.00194 28555124 PMC5431013

[29] Melendez-Calderon A Shirota C Balasubramanian S. Estimating movement smoothness from inertial measurement units. Front Bioeng Biotechnol. (2021) 8:1–16. 10.3389/fbioe.2020.558771 33520949 PMC7841375

[30] do Vale Garcia F da Cunha MJ Schuch CP Schifino GP Balbinot G Pagnussat AS. Movement smoothness in chronic post-stroke individuals walking in an outdoor environment—a cross-sectional study using IMU sensors. PLoS ONE. (2021) 16:1–18. 10.1371/journal.pone.0250100 33886640 PMC8061986

[31] Iijima H Eguchi R Aoyama T Takahashi M. Trunk movement asymmetry associated with pain, disability, and quadriceps strength asymmetry in individuals with knee osteoarthritis: a cross-sectional study. Osteoarthrit Cartilage. (2019) 27:248–56. 10.1016/j.joca.2018.10.012 30445222

[32] Beck Y Herman T Brozgol M Giladi N Mirelman A Hausdorff JM. SPARC: a new approach to quantifying gait smoothness in patients with Parkinson's disease. J NeuroEng Rehabil. (2018) 15:1–9. 10.1186/s12984-018-0398-3 29914518 PMC6006701

[33] Pinto C Schuch CP Balbinot G Salazar AP Hennig EM Kleiner AFR. Movement smoothness during a functional mobility task in subjects with Parkinson's disease and freezing of gait—an analysis using inertial measurement units. J NeuroEng Rehabil. (2019) 16:8. 10.1186/s12984-019-0579-8 31488184 PMC6729092

[34] Figueiredo AI Balbinot G Brauner FO Schiavo A Baptista RR Pagnussat AS. SPARC metrics provide mobility smoothness assessment in oldest-old with and without a history of falls: a case control study. Front Physiol. (2020) 11:540. 10.3389/fphys.2020.00540 32587523 PMC7298141

[35] Balasubramanian S Melendez-Calderon A Roby-Brami A Burdet E. On the analysis of movement smoothness. J NeuroEng Rehabil. (2015) 12:9. 10.1186/s12984-015-0090-9 26651329 PMC4674971

[36] Gage H. Accelerographic analysis of human gait. Am Society Mechanical Engineers. In: Biomechanics Monograph (1967). p. 137–52.

[37] Pasciuto I Bergamini E Iosa M Vannozzi G Cappozzo A. Overcoming the limitations of the Harmonic Ratio for the reliable assessment of gait symmetry. J Biomech. (2017) 53:84–9. 10.1016/j.jbiomech.2017.01.005 28104246

[38] Bellanca JL Lowry KA VanSwearingen JM Brach JS Redfern MS. Harmonic ratios: a quantification of step to step symmetry. J Biomech. (2013) 46:828–31. 10.1016/j.jbiomech.2012.12.008 23317758 PMC4745116

[39] Henriksen M Lund H Moe-Nilssen R Bliddal H Danneskiod-Samsøe B. Test-retest reliability of trunk accelerometric gait analysis. Gait Post. (2004) 19:288–97. 10.1016/S0966-6362(03)00069-9 15125918

[40] Mansour KB Gorce P Rezzoug N. The multifeature gait score: an accurate way to assess gait quality. PLoS ONE. (2017) 12:1–12. 10.1371/journal.pone.0185741 29049403 PMC5648116

[41] Shrout PE Fleiss JL. Intraclass correlations: uses in assessing rater reliability. Psychol Bullet. (1979) 86:420–8. 18839484 10.1037//0033-2909.86.2.420

[42] Bobak CA Barr PJ O'Malley AJ. Estimation of an inter-rater intra-class correlation coefficient that overcomes common assumption violations in the assessment of health measurement scales. BMC Med Res Methodol. (2018) 18:6. 10.1186/s12874-018-0550-6 30208858 PMC6134634

[43] Stellmann JP Neuhaus A Götze N Briken S Lederer C Schimpl M. Ecological validity of walking capacity tests in multiple sclerosis. PLoS ONE. (2015) 10:e0123822. 10.1371/journal.pone.0123822 25879750 PMC4399985

[44] Carcreff L Gerber CN Paraschiv-Ionescu A De Coulon G Newman CJ Aminian K. Comparison of gait characteristics between clinical and daily life settings in children with cerebral palsy. Sci Rep. (2020) 10:2091. 10.1038/s41598-020-59002-6 32034244 PMC7005861

[45] Del Din S Godfrey A Galna B Lord S Rochester L. Free-living gait characteristics in ageing and Parkinsons disease: impact of environment and ambulatory bout length. J NeuroEng Rehabil. (2016) 13:46. 10.1186/s12984-016-0154-5 27175731 PMC4866360

[46] Manji H Connolly S Kitchen N Lambert C Mehta A. Oxford Handbook of Neurology. 2 ed. New York, NY: Oxford University Press (2014).

[47] Comber L Galvin R Coote S. Gait and Posture Gait de fi cits in people with multiple sclerosis : a systematic review and. Gait Post. (2017) 51:25–35. 10.1016/j.gaitpost.2016.09.026 27693958

[48] Preiningerova JL Novotna K Rusz J Sucha L Ruzicka E Havrdova E. Spatial and temporal characteristics of gait as outcome measures in multiple sclerosis (EDSS 0 to 6.5). J Neuroeng Rehabil. (2015) 12:15. 10.1186/s12984-015-0001-0 25890382 PMC4334845

[49] Bois A Tervil B Moreau A Vienne-Jumeau A Ricard D Oudre L. A topological data analysis-based method for gait signals with an application to the study of multiple sclerosis. PLoS ONE. (2022) 17:e0268475. 10.1371/journal.pone.0268475 35560328 PMC9106173

[50] Bruijn SM van Dieën JH Meijer OG Beek PJ. Is slow walking more stable? J Biomech. (2009) 42:1506–12. 10.1016/j.jbiomech.2009.03.047 19446294

[51] CofrLizama LE Bruijn SM Galea MP. Gait stability at early stages of multiple sclerosis using different data sources. Gait Post. (2020) 77:214–7. 10.1016/j.gaitpost.2020.02.006 32058286

[52] Alster SD Menascu S Achiron A Kalron A Dolev M Givon U. Longitudinal relationships between disability and gait characteristics in people with MS. Sci Rep. (2022) 12:3653. 10.1038/s41598-022-07734-y 35256705 PMC8901766

[53] Givon U Zeilig G Achiron A. Gait analysis in multiple sclerosis: characterization of temporal-spatial parameters using GAITRite functional ambulation system. Gait Post. (2009) 29:138–42. 10.1016/j.gaitpost.2008.07.011 18951800

[54] Buckley C Micó-Amigo ME Dunne-Willows M Godfrey A Hickey A Lord S. Gait asymmetry post-stroke: determining valid and reliable methods using a single accelerometer located on the trunk. Sensors. (2020) 20:1–17. 10.3390/s20010037 31861630 PMC6983246

[55] Gulde P Hermsdörfer J Rieckmann P. Speed but not smoothness of gait reacts to rehabilitation in multiple sclerosis. Mult Scler Int. (2021) 2021:1–8. 10.1155/2021/5589562 34123427 PMC8192191

[56] Bohannon RW Glenney SS. Minimal clinically important difference for change in comfortable gait speed of adults with pathology: a systematic review. J Eval Clin Pract. (2014) 20:295–300. 10.1111/jep.12158 24798823

