# Supplementary material for: Innovative multidimensional gait evaluation using IMU in multiple sclerosis: introducing the semiogram
Source: Front Neurol. 2023 Sep 15;14:1237162. doi: 10.3389/fneur.2023.1237162 (PMC10540441; doi:10.3389/fneur.2023.1237162)
Supplement: Supplementary file 1 [file Data_Sheet_1.PDF]

Supplementary Material

1 SUPPLEMENTARY DATA

Supplementary Material should be uploaded separately on submission. Please include any supplementary data, figures and/or tables. All supplementary files are deposited to FigShare for permanent storage and receive a DOI.

Supplementary material is not typeset so please ensure that all information is clearly presented, the appropriate caption is included in the file and not in the manuscript, and that the style conforms to the rest of the article. To avoid discrepancies between the published article and the supplementary material, please do not add the title, author list, affiliations or correspondence in the supplementary files.

2 SUPPLEMENTARY TABLES AND FIGURES

2.1 Figures

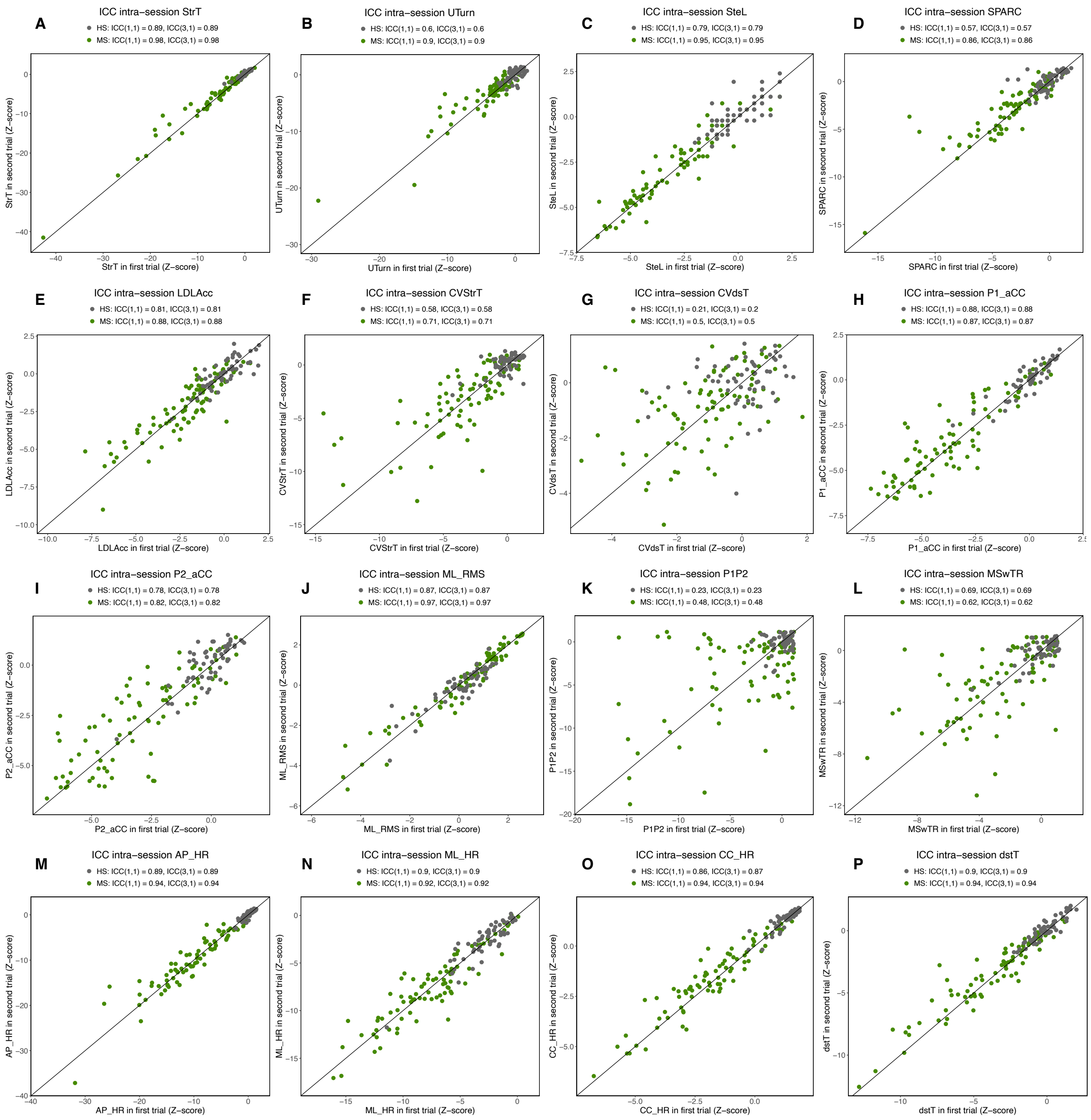

Figure S1. Intra-session intraclass correlation coefficients (ICCs) for the qualitative parameters included in the semiogram. **A**: stride time; **B**: U-turn time; **C**: step length; **D**: spectral arc length computed from the trunk gyration; **E**: log-dimensionless jerk computed from the trunk acceleration; **F**: coefficient of variation of the stride time; **G**: coefficient of variation of the double stance time; **H**: step autocorrelation coefficient of the trunk craniocaudal acceleration; **I**: stride autocorrelation coefficient of the trunk craniocaudal acceleration; **J**: root mean square of the trunk mediolateral acceleration; **K**: ratio P1 to P2; **L**: ratio of left and right swing times; **M**: improved harmonic ratio of the trunk anteroposterior acceleration; **N**: improved harmonic ratio of the trunk mediolateral acceleration; **O**: improved harmonic ratio of the trunk craniocaudal acceleration; **P**: double stance time.

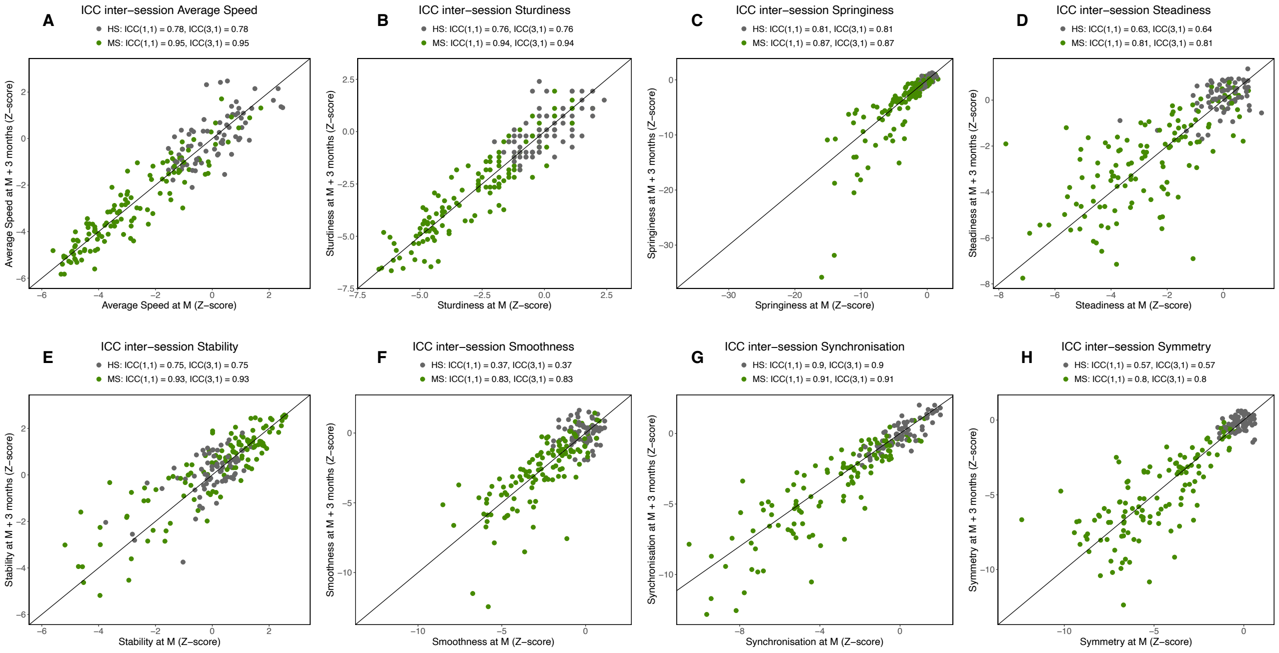

Figure S2. Inter-session ICCs. For speed (**A**) and the seven criteria for the semiogram: sturdiness (**D**), stability (**E**), synchronization (**F**), smoothness (**G**), symmetry (**H**). ICCs are reported for the two populations separately: progressive multiple sclerosis (pMS) and healthy subjects (HS). ICC(1,1) supposes that subject variability is due to measurement error, and ICC(3,1) supposes that subject variability is due to systematic bias.

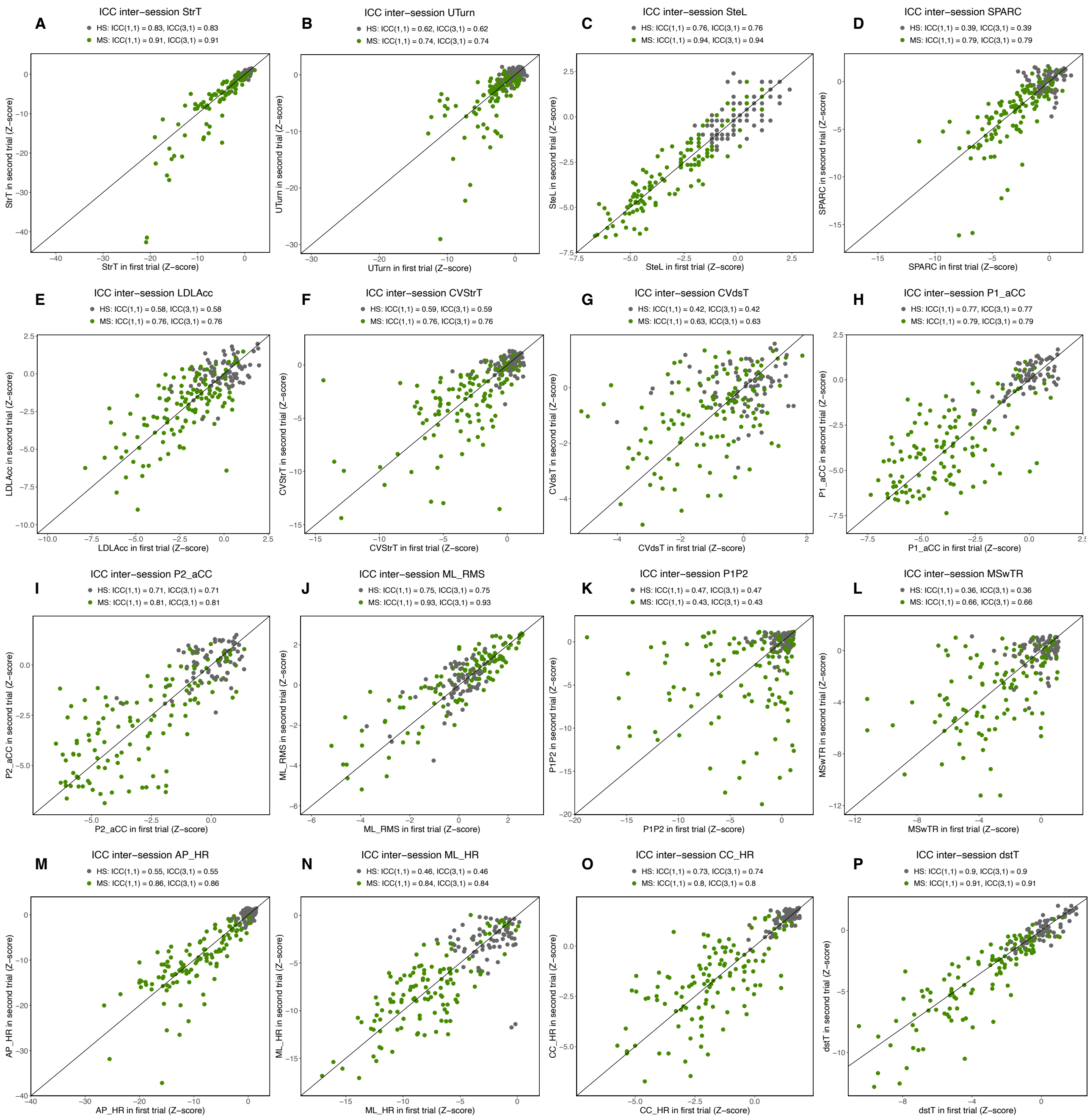

Figure S3. Inter-session intraclass correlation coefficients (ICCs) for the qualitative parameters included in the semiogram. **A**: stride time; **B**: U-turn time; **C**: step length; **D**: spectral arc length computed from the trunk gyration; **E**: log-dimensionless jerk computed from the trunk acceleration; **F**: coefficient of variation of the stride time; **G**: coefficient of variation of the double stance time; **H**: step autocorrelation coefficient of the trunk craniocaudal acceleration; **I**: stride autocorrelation coefficient of the trunk craniocaudal acceleration; **J**: root mean square of the trunk mediolateral acceleration; **K**: ratio P1 to P2; **L**: ratio of left and right swing times; **M**: improved harmonic ratio of the trunk anteroposterior acceleration; **N**: improved harmonic ratio of the trunk mediolateral acceleration; **O**: improved harmonic ratio of the trunk craniocaudal acceleration; **P**: double stance time.

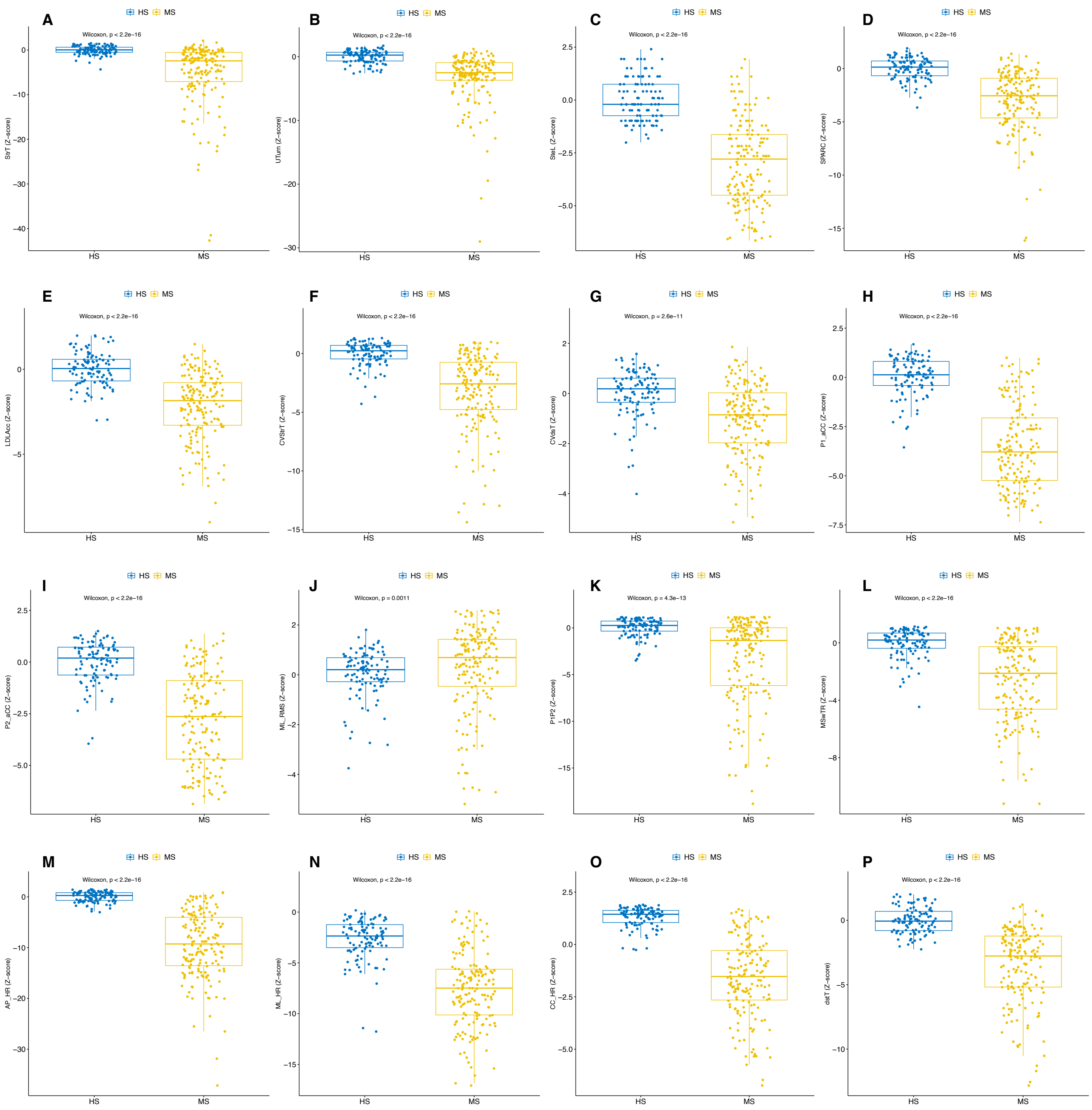

Figure S4. Difference in each qualitative parameter of the semiogram between the two populations (HS and MS). A: stride time; **B**: U-turn time; **C**: step length; **D**: spectral arc length computed from the trunk gyration; **E**: log-dimensionless jerk computed from the trunk acceleration; **F**: coefficient of variation of the stride time; **G**: coefficient of variation of the double stance time; **H**: step autocorrelation coefficient of the trunk craniocaudal acceleration; **I**: stride autocorrelation coefficient of the trunk craniocaudal acceleration; **J**: root mean square of the trunk mediolateral acceleration; **K**: ratio P1 to P2; **L**: ratio of left and right swing times; **M**: improved harmonic ratio of the trunk anteroposterior acceleration; **N**: improved harmonic ratio of the trunk mediolateral acceleration; **O**: improved harmonic ratio of the trunk craniocaudal acceleration; **P**: double stance time.
